# Transient Changes of Metabolism at the Pronuclear Stage in Mice Influences Skeletal Muscle Phenotype in Adulthood

**DOI:** 10.3390/ijms21197203

**Published:** 2020-09-29

**Authors:** Christelle Bertrand-Gaday, Martine Letheule, Emilie Blanchet, Barbara Vernus, Laurence Pessemesse, Amélie Bonnet-Garnier, Anne Bonnieu, François Casas

**Affiliations:** 1 DMEM, Univ. Montpellier, INRAE, 34060 Montpellier, France; christelle.bertrand-gaday@inrae.fr (C.B.-G.); emilie.blanchet@inrae.fr (E.B.); barbara.vernus@inrae.fr (B.V.); laurence.pessemesse@inrae.fr (L.P.); anne.bonnieu@inrae.fr (A.B.); 2 Université Paris-Saclay, UVSQ, INRAE, BREED, 78350 Jouy-en-Josas, France; martine.letheule@inrae.fr (M.L.); amelie.bonnet-garnier@inrae.fr (A.B.-G.); 3 Ecole Nationale Vétérinaire d’Alfort, BREED, 94700 Maisons-Alfort, France

**Keywords:** embryo, metabolic reprogramming, mitochondria, skeletal muscle, epigenetic

## Abstract

Skeletal muscle has a remarkable plasticity, and its phenotype is strongly influenced by hormones, transcription factors, and physical activity. However, whether skeletal phenotype can be oriented or not during early embryonic stages has never been investigated. Here, we report that pyruvate as the only source of carbohydrate in the culture medium of mouse one cell stage embryo influenced the establishment of the muscular phenotype in adulthood. We found that pyruvate alone induced changes in the contractile phenotype of the skeletal muscle in a sexually dependent manner. For male mice, a switch to a more glycolytic phenotype was recorded, whereas, in females, the pyruvate induced a switch to a more oxidative phenotype. In addition, the influence of pyruvate on the contractile phenotypes was confirmed in two mouse models of muscle hypertrophy: the well-known myostatin deficient mouse (Mstn−/−) and a mouse carrying a specific deletion of p43, a mitochondrial triiodothyronine receptor. Finally, to understand the link between these adult phenotypes and the early embryonic period, we assessed the levels of two histone H3 post-translational modifications in presence of pyruvate alone just after the wave of chromatin reprogramming specific of the first cell cycle. We showed that H3K4 acetylation level was decreased in Mstn−/− 2-cell embryos, whereas no difference was found for H3K27 trimethylation level, whatever the genotype. These findings demonstrate for the first time that changes in the access of energy substrate during the very first embryonic stage can induce a precocious orientation of skeletal muscle phenotype in adulthood.

## 1. Introduction

Skeletal muscle is known for its remarkable plasticity and its capacity to respond to environmental and physiological challenges by changing its phenotype in terms of size, composition, and metabolic properties. In particular, muscle fiber phenotype is strongly influenced by hormones, transcription factors, physical activity, and the motor neurons. During the last few decades, numerous models of transgenic mice demonstrated the role of several proteins in the control of muscle mass and the metabolic and contractile features of muscle fibers. Among them, myostatin (Mstn), a member of the transforming growth factor-β superfamily, is the most potent inhibitor of skeletal muscle mass. Disruption of the myostatin gene in mice (Mstn−/−) results in a dramatic increase of muscle mass and to an overall glycolytic muscle phenotype [1]. In our team, we have previously identified a 43 kDa truncated form of the nuclear thyroid hormone receptor alpha 1 TRα1 (p43) which is synthesized by the use of internal initiation sites of translation presents in the TRα1 transcript [2]. Despite the occurrence of a nuclear localization signal, p43 is specifically imported into the mitochondria and binds specific sequences of the mitochondrial genome, sharing strong homologies with nuclear T3 Responsive Element (T3RE) [3,4,5]. We also showed that specific overexpression of p43 in mouse skeletal muscle (p43-Tg) increased mitochondrial respiration [6]. Conversely, depletion of p43 in mice (p43−/−) reduced the activity of the mitochondrial respiratory chain [7]. These changes in mitochondrial activity induced in these mice a change in the muscle contractile phenotype. Thus, the overexpression of p43 promotes the expression of slower and more oxidative muscle fibers [6], and, on the contrary, the absence of p43 promotes the expression of faster and more glycolytic muscle fibers [7]. We also observed, in p43−/−, a moderate increase of muscle mass, whereas a decrease is found in p43-Tg mice [8]. Lastly, we demonstrated that p43 controls the duration of skeletal muscle regeneration [9]. Fertilization and early stages of development in mammals are highly sensitive to metabolic perturbations [10], and evidence indicates that composition of embryo culture media may adversely affect the developmental potential and overall post-natal health of the organism according to the Developmental Origins of Health and Disease (DOHaD) hypothesis [11]. Several animal studies have reported that nutritional, oxidative, and in vitro stresses during the preimplantation development period are sufficient to alter developmental growth and metabolism [12,13,14,15,16,17]. In particular, recent studies revealed that small changes in the composition of carbohydrate substrates (glucose, pyruvate, and lactate), amino acids, pH, and pO_2_ in the early stages of development can modify the metabolic activity of embryos and determine their fate, including glucose metabolism and vascular function [12,18,19,20,21,22,23]. In addition, sexual dimorphism effects were also observed [18,24]. These observations underline that fertilization and early stages of development in mammals are highly sensitive to metabolism functioning. This suggests that common mechanisms may be involved in the detection and transduction of environmental stimuli, which could induce changes in epigenetic, transcriptional and/or metabolic reprogramming [25,26]. The degree of DNA compaction that controls the accessibility of specific genetic loci is notably regulated by histones [27]. The major histone post-translational modifications are acetylation and methylation, but others possibilities exist (phosphorylation, ubiquitination, SUMOylation) [28]. Each of these modifications can elicit positive or negative effects on DNA compaction and genes accessibility which allow transcriptional activation or repression [29,30]. However, the question of whether the skeletal phenotype could be oriented during the early embryonic stage has never been investigated. To explore this question under experimental conditions, we chose to induce a transient NAD(P)H oxidation as early as the pronuclear stage (PN) by changing the medium composition as previously described [12]. For this purpose, we compared M16 medium which contains pyruvate, lactate, and glucose as carbohydrate energy source and a M16/pyruvate medium containing pyruvate as the only source of carbohydrate which is known to stimulate mitochondrial activity [12,31]. 

We report here that the establishment of the muscular phenotype in adulthood is highly sensitive to carbohydrate composition of the medium at the PN stage. Moreover, this observation was confirmed using two mouse models of muscle hypertrophy (Mstn−/− and p43−/−). In addition, the changes in the contractile phenotype of the skeletal muscle were gender dependent. For all male genotypes, a switch to a more glycolytic phenotype was recorded, whereas, in females, the pyruvate induced a switch to a more oxidative phenotype. This study demonstrated, for the first time, the potential role of pyruvate in culture media as early as the PN stage to induce precocious orientation of skeletal muscle phenotype in adulthood. To study one of the mechanisms involved in the transduction of this metabolic change, we analyzed the level of two epigenetic marks just after the PN stage. Although no differences were revealed for H3K27me3, a slight decrease was observed for H3K4ac. Our results suggest that these experimental conditions could be an opportunity to understand the mechanisms, for example, epigenetic, involved in the detection and transduction of environmental input into a programmed metabolic response.

## 2. Results

### 2.1. Experimental Design 

**Figure 1:**
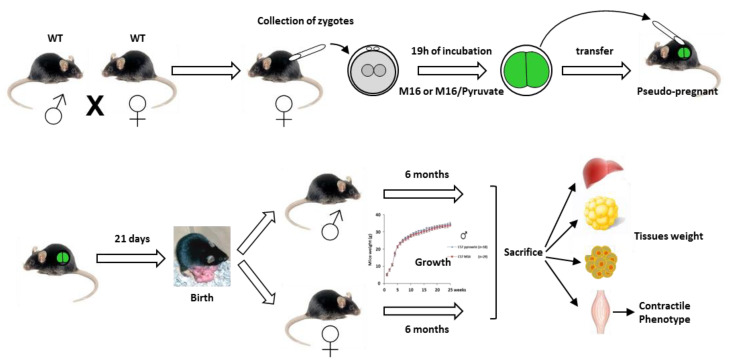
Experimental design and developmental potential. WT: Wild Type.

In this study, we chose to collect the 1-cell embryos after natural mating to avoid possible biases related to superovulation. After collection, fertilized oocytes from control mice (Wild Type (WT)) were incubated for about 19 h in a standard M16 culture medium containing pyruvate, lactate, and glucose as energy source, or containing pyruvate as the only energy substrate (M16/pyruvate), in order to induce NAD(P)H oxidation and stimulate mitochondrial activity, as previously described [12]. The 2-cell embryos were then transferred to pseudo-pregnant recipients, and 90 newborns were weighed every week for 6 months (Figure 1) (Figure S1A). We observed that the cleavage rate and the percentage of birth following embryo transfer in 1-cell embryo cultured in M16/pyruvate were similar to those obtained previously (respectively, 90% and 60%) [12] (Figure S1A). In addition, we have compared the litter size of the two experimental groups with naturally mated mice (in vivo), and we found that the litter size of C57Bl/6J mice in the in vivo group was significantly larger than that of manipulated embryos (7 vs. 5 for M16 and 7 vs. 5.6 for M16/pyruvate) (Figure 1). Post-natal growth profiles of animals of both genders were then recorded until 6 months for the two experimental groups. 

### 2.2. Post-Natal Growth of C57Bl6 Mice Was Not Affected by a Short Exposure in M16/Pyruvate Medium at 1 Cell Embryo Stage. Wild Type (WT)

**Figure 2:**
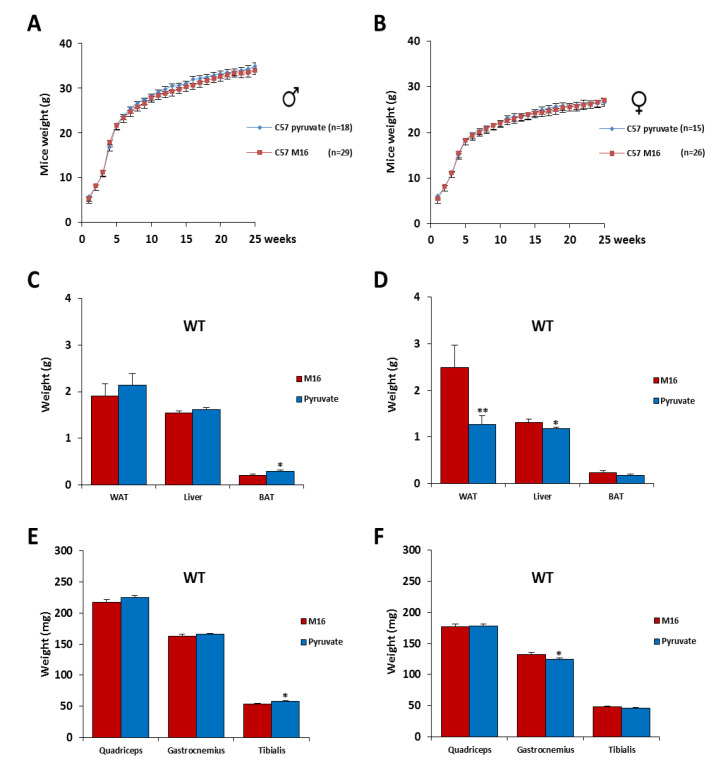
Post-natal growth of C57Bl/6J mice was not affected by a short exposure in M16/pyruvate medium at 1-cell embryo stage. Post-natal growth profiles of male (**A**) and female (**B**) C57Bl/6J mice derived from zygotes incubated in M16 or M16/pyruvate media. At 6 months of age, the mice were sacrificed, and white adipose tissue (WAT), brown adipose tissue (BAT), and liver were weighed (**C**): male; (**D**): female, as well as the skeletal muscles (quadriceps, gastrocnemius, and tibialis) (**E**): male; (**F**): female. For male C57Bl/6J mice, *n* = 18 in M16 medium and 29 in M16/pyruvate medium. For female C57Bl/6J mice, *n* = 15 in M16 medium and 26 in M16/pyruvate medium. Statistical significance: * *p* < 0.05, ** *p* < 0.01, Student’s *t*-test. Results are expressed as ±sem.

We observed that post-natal growth until 6 months of C57Bl/6J mice of both genders was similar in the two experimental groups (M16 vs. M16/pyruvate) (Figure 2A,B). At 6 months of age, the mice were sacrificed and several organs and tissues were weighed. In C57Bl/6J male mice, we found an increase of brown adipose tissue (BAT) (+42%, *p* < 0.05) and tibialis muscle (+8%, *p* < 0.05) weight in the group of animals derived from zygotes incubated in M16/pyruvate medium compared to those incubated in M16 medium, whereas no differences were recorded for the white adipose tissue (WAT) and liver nor for large muscles, such as quadriceps and gastrocnemius (Figure 2C,E). In C57Bl/6J female mice, we observed significant decrease of the WAT (−49%, *p* < 0.01), liver (−10%, *p* < 0.05), and gastrocnemius muscle (−6%, *p* < 0.05) weight in the M16/pyruvate group, whereas the BAT, quadriceps, and tibialis remained unaffected (Figure 2D,F).

### 2.3. The Contractile Phenotype of the Skeletal Muscle is Highly Sensitive to a Short Exposure in M16/Pyruvate Medium at 1-Cell Embryo Stage

**Figure 3:**
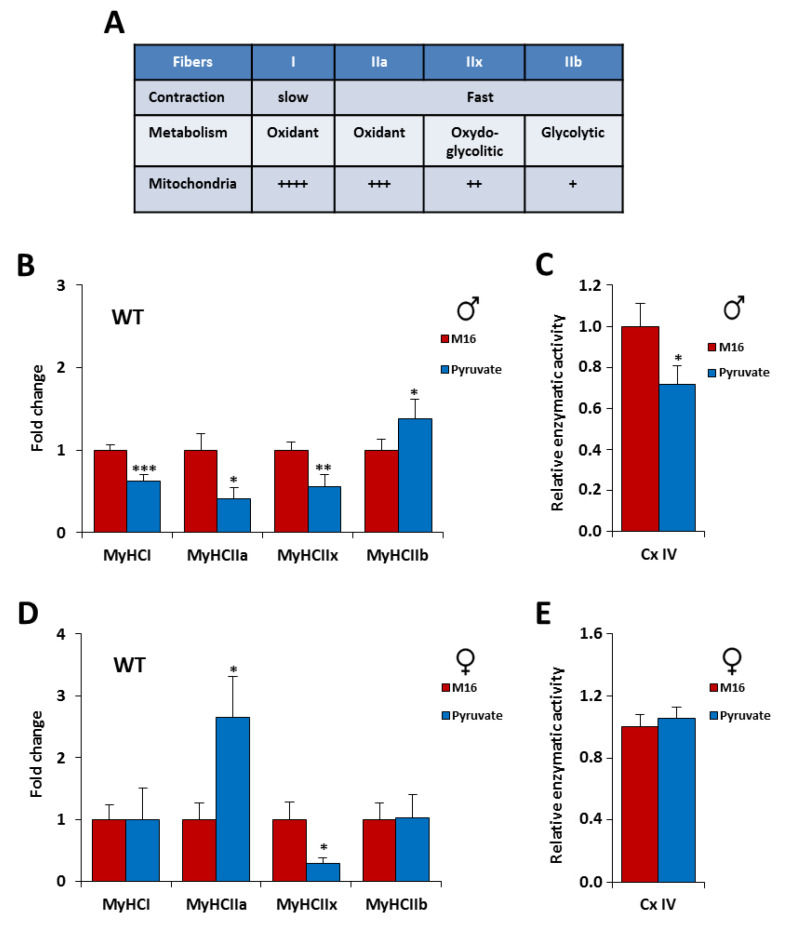
The contractile phenotype of the skeletal muscle of C57Bl/6J mice is highly sensitive to a short exposure in M16/pyruvate medium at the 1 cell embryo stage. (**A**) Contractile and metabolic characteristics of muscle fibers. Mitochondria content: ++++ (very high), +++ (high), ++ (medium high) and + (low). Relative mRNA expression levels of the four Myosin Heavy Chain (MyHC) isoforms in tibialis muscle of male (**B**) and female (**D**) C57Bl/6J mice derived from zygotes incubated in M16 or M16/pyruvate media at 6 months of age (*n* = 8 each group). Mitochondrial complex IV activity (cytochrome c oxidase) in tibialis muscle of male (**C**) and female (**E**) C57Bl/6J mice derived from zygotes incubated in M16 or M16/pyruvate media at 6 months of age (*n* = 8 each group). Values obtained are expressed in percent of the corresponding control value. Statistical significance: * *p* < 0.05, ** *p* < 0.01, *** *p* < 0.001, Student’s *t*-test. Results are expressed as ±sem.

Skeletal muscle contains muscle fibers that have different metabolic and contractile characteristics. Slow-twitch fibers express the myosin heavy chain (MyHC) type I and have a high mitochondrial density and a high oxidative metabolism. Fast-twitch fibers express MyHC type II, including 3 subtypes: IIa, IIx, and IIb. IIa fibers have a high mitochondrial density and a high oxidative metabolism, whereas IIb fibers exhibit a low mitochondrial density and have a high glycolytic metabolism. IIx fibers have an oxidative capacity intermediate between that of type IIa and IIb [32,33] (Figure 3A). To better characterize the influence of transient exposure of zygote to M16 or M16/pyruvate media on contractile skeletal muscle phenotype, we measured the expression of the 4 adult myosin heavy chains (MyHC) by quantitative PCR on Tibilias, an oxido-glycolytic muscle. The proportion of muscle fibers in the tibialis is as follows: IIb (50%), IIx (35%), IIa (14%), and I (1%) [34].

The study of the influence of transient exposure to M16 and M16/pyruvate at fertilization revealed interesting data. First, in the tibialis muscle we observed for C57Bl/6J male mice a switch to a more glycolytic phenotype. Indeed, we recorded, in the M16/pyruvate condition, a decreased MyHC expression of type I (−37%, *p* < 0.001), IIa (−59%, *p* < 0.01) and IIx (−46%, *p* < 0.05) and an increased expression of MyHC IIb (+38%, *p* < 0.05%) (Figure 3B). Surprisingly, in C57Bl/6J female mice an opposite switch to a more oxidative phenotype was shown. We found in M16/pyruvate condition, an increase expression of MyHC of type IIa (+165%, *p* < 0.05) and a decrease expression of MyHC IIx (−71%, *p* < 0.05) compared to M16 (Figure 3D). To decipher whether these changes in the contractile phenotype could influence the metabolic capacity of the whole muscle, we studied the activity of the complex IV of the mitochondrial respiratory chain (cytochrome c oxidase) in tibialis. In male C57Bl/6J mice, the M16/pyruvate medium induced a significant decrease in the activity of the mitochondrial IV complex which is consistent with the drop in the expression of type I, IIa, and IIb fibers observed previously (Figure 3C). However, in female C57Bl/6J mice, the increase in the number of IIa fibers to the detriment of the number of IIx fibers was not sufficient to induce a change in mitochondrial activity (Figure 3E). Taken together, these data indicate that the contractile phenotype of the skeletal muscle was highly sensitive to pyruvate as the only source of carbohydrate in the culture medium. In addition, we revealed that the phenotype is gender dependent.

These observations led us to ask what would happen if we used transgenic mouse models of muscle hypertrophy. Can the hypertrophy observed in our animal models be accentuated or, conversely attenuated? To answer these questions two mouse models of muscle hypertrophy were chosen: Mstn−/− and p43−/− mice. Myostatin deficiency results in a very large increase of muscle mass in mice associated with a very glycolytic metabolism [1]. P43 deficient mice exhibit reduced mitochondrial activity in muscle, a change in metabolic and contractile features of the muscle fibers, but also a moderate increase of muscle mass (+14%) [7]. This last model was chosen because the absence of p43 leads to an inhibition of mitochondrial activities and which would antagonize the pyruvate effect which induces NAD(P)H oxidation and stimulates the activity of the organelle [12,19].

### 2.4. Skeletal Muscle Hypertrophy Was Fully Abrogated for p43−/− Male Mice by a Short Exposure in M16/Pyruvate Medium at 1 Cell Embryo Stage

**Figure 4:**
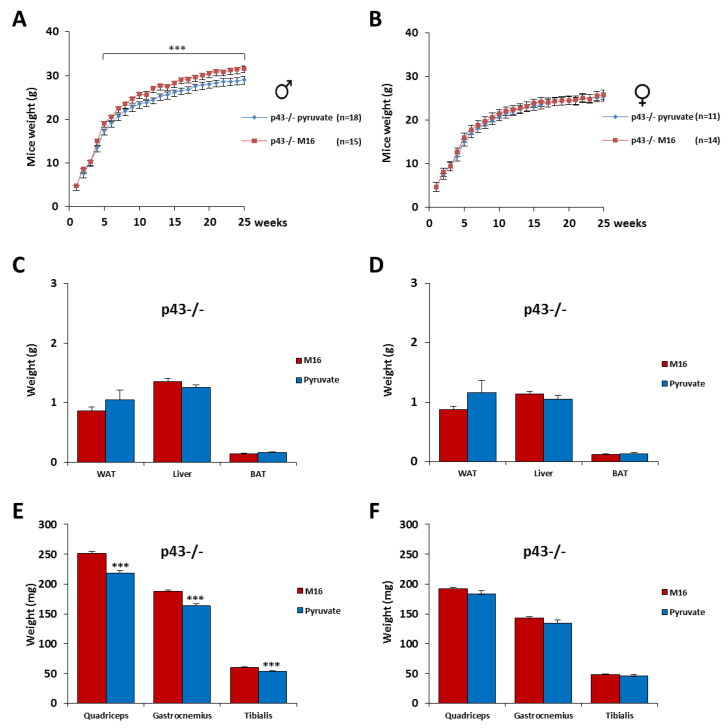
Skeletal muscle hypertrophy was fully abrogated for p43−/− male mice by a short exposure in M16/pyruvate medium at the 1 cell embryo stage. Post-natal growth profiles of male (**A**) and female (**B**) p43−/− mice derived from zygotes incubated in M16 or M16/pyruvate media. At 6 months of age, the mice were sacrificed and WAT, BAT, and liver were weighed (**C**: male; **D**: female), as well as the skeletal muscles (quadriceps, gastrocnemius, and tibialis) (**E**: male; **F**: female). For male p43−/− mice, *n* = 18 in M16 medium and 15 in M16/pyruvate medium. For female p43−/− mice, *n* = 11 in M16 medium and 14 in M16/pyruvate medium. Statistical significance: *** *p* < 0.001, Student’s *t*-test. Results are expressed as ±sem.

For the mice devoid of p43, the post-natal growth profiles of 58 animals of both genders were recorded until 6 months for the two experimental groups. As previously described [7] we found that p43−/− mice are leaner, less fat, and have more muscle mass than C57Bl/6J mice (Figure 2 and Figure 3). Males from 1 month derived from zygotes incubated in M16/pyruvate medium were significantly lighter than those obtained with M16 medium (Figure 4A). In line with this fall of total body weight, we found, in these p43−/− male mice, a strong decrease of the quadriceps (−13%, *p* < 0.001), gastrocnemius (−13%, *p* < 0.001), and the tibialis (−11%, *p* < 0.001) muscles weight compared to those incubated in M16 medium, whereas no difference of weight were recorded for the WAT, BAT, and liver (Figure 4C,E). Overall, it is important to note that the skeletal muscle hypertrophy described in p43−/− male mice was fully abrogated by the short exposure of the zygotes in M16/pyruvate medium. In contrast, the post-natal growth and the weight of the organs and tissues were similar in p43−/− female mice of the two groups (Figure 4B,D,F). 

### 2.5. Skeletal Muscle Hypertrophy Was Increased in Mstn−/− Mice of Both Genders by a Short Exposure in M16/Pyruvate Medium at 1 Cell Embryo Stage

**Figure 5:**
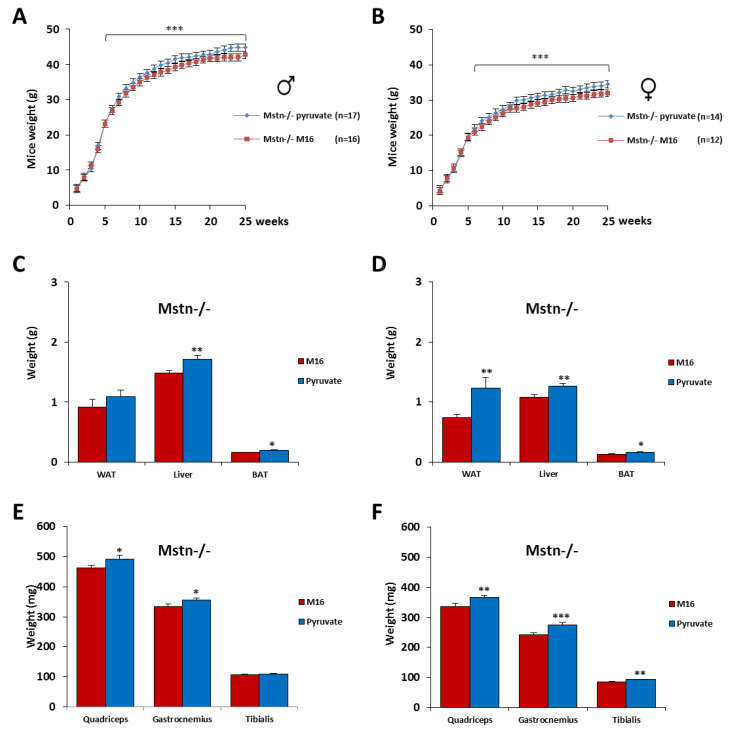
Skeletal muscle hypertrophy was increased in myostatin deficient (Mstn−/−) mice of both genders by a short exposure in M16/pyruvate medium at 1 cell embryo stage. Post-natal growth profiles of male (**A**) and female (**B**) Mstn−/− mice derived from zygotes incubated in M16 or M16/pyruvate media. At 6 months of age, the mice were sacrificed, and WAT, BAT, and liver were weighed (**C**): male; (**D**): female, as well as the skeletal muscles (quadriceps, gastrocnemius, and tibialis) (**E**): male; (**F**): female. For male Mstn−/− mice, *n* = 17 in M16 medium and 16 in M16/pyruvate medium. For female Mstn−/− mice, *n* = 14 in M16 medium and 12 in M16/pyruvate medium. Statistical significance: * *p* < 0.05, ** *p* < 0.01, *** *p* < 0.001, Student’s *t*-test. Results are expressed as ±sem.

The post-natal growth profiles of 59 animals devoid of myostatin of both genders were recorded until 6 months for the two experimental groups. As previously described [1] we found that Mstn−/− mice are leaner and less fat than C57Bl/6J mice and also present a stronger muscle mass than p43−/− and C57Bl/6J mice (Figures S2 and S3). Mstn−/− mice from 7 weeks of both genders derived from zygotes incubated in M16/pyruvate medium became significantly heavier (Figure 5A,B). In Mstn−/− male mice, we found an increase of liver (+16%, *p* < 0.01) and BAT (+23%, *p* < 0.05) weight, as well as quadriceps (+6%, *p* < 0.05) and gastrocnemius (+7%, *p* < 0.05%) muscles, in the group of animals derived from zygotes incubated in M16/pyruvate medium compared to those incubated in M16 medium (Figure 5C,E). In Mstn−/− female mice, we recorded an increase of liver (+18%, *p* < 0.01), WAT (+66%, *p* < 0.01), and BAT (+26%, *p* < 0.05) weight, as well as quadriceps (+9%, *p* < 0.01), gastrocnemius (+13%, *p* < 0.001), and the tibialis (+10%, *p* < 0.01) muscles, compared to those incubated in M16 medium (Figure 5D,F). Overall, our data demonstrated that the skeletal muscle hypertrophy in Mstn−/− mice was further increased by the short exposure of the zygotes to M16/pyruvate medium.

### 2.6. Short Exposure in M16/Pyruvate Medium at 1 Cell Embryo Stage Also Alters Contractile Features of Skeletal Muscle in p43−/− and Mstn−/− Mice

**Figure 6:**
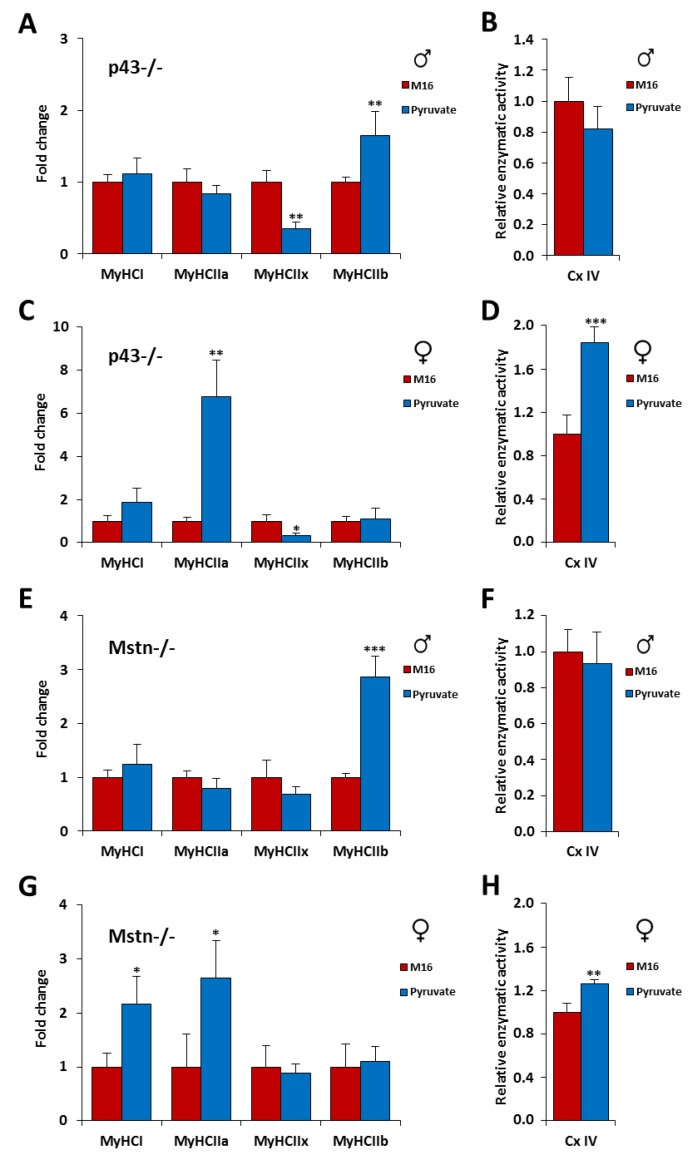
Short exposure in M16/pyruvate medium at 1 cell embryo stage also alters contractile features of skeletal muscle in p43−/− and Mstn−/− mice. Relative mRNA expression levels of the four Myosin Heavy Chain (MyHC) isoforms in tibialis muscle of male (**A**,**E**) and female (**C**,**G**) p43−/− and Mstn−/− mice derived from zygotes incubated in M16 or M16/pyruvate media at 6 months of age (*n* = 8 each group). Mitochondrial complex IV activity (cytochrome c oxidase) in tibialis muscle of male (**B**,**F**) and female (**D**,**H**) p43−/− and Mstn−/− mice derived from zygotes incubated in M16 or M16/pyruvate media at 6 months of age (*n* = 8 each group). Values obtained are expressed in percent of the corresponding control value. Statistical significance: * *p* < 0.05, ** *p* < 0.01, *** *p* < 0.001, Student’s *t*-test. Results are expressed as ±sem.

Our results show that the skeletal muscle mass can be affected by the pyruvate contained in the culture medium depending on the genotype and the sex. However, what about contractile characteristics? As previously shown [1,7], p43−/− and Mstn−/− mice have a more glycolytic muscle phenotype than C57Bl/6J mice with less oxidative fibers (I, IIa, and IIx) and more glycolytic fibers (IIb) (Figure S4). In addition, mitochondrial complex IV activity is also reduced in tibialis muscle of p43−/− and Mstn−/− mice compared to C57Bl/6J animals (Figure S4). As observed previously for C57Bl/6J male mice, in the M16/pyruvate condition, we found a switch for a more glycolytic phenotype in males of our mouse models of hypertrophy (Figure 6A,E). Thus, with the M16/pyruvate condition, in p43−/− muscles we showed a decreased expression of MyHC of type IIx (−64%, *p* < 0.01) and an increased expression of MyHC IIb (+64%, *p* < 0.01). For Mstn−/− muscles which are already highly glycolytic the proportion of MyHC IIb (+186%, *p* < 0.001) increases further (Figure 6A–E). In line with our data obtained for C57Bl/6J female mice, an opposed switch for a more oxidative phenotype was shown for the transgenic animals (Figure 6B–D). Thus, in the p43−/− female mice, in the M16/pyruvate condition, we recorded an increased expression of MyHC of type IIa (+575%, *p* < 0.01) and a decrease expression of MyHC IIx (−66%, *p* < 0.05), and, for Mstn−/− female mice, we found an increased expression of MyHC of type I (+117%, *p* < 0.001) and IIa (+165%, *p* < 0.001) (Figure 6C,G). In addition, we showed that the increase of the proportion of MyHC IIb in tibialis muscle of p43−/− and Mstn−/− male mice, which are already highly glycolytic, observed in the M16/pyruvate condition, did not significantly influence the activity of the mitochondrial complex IV (Figure 6B,F). However, our data revealed that the increase of the proportion of MyHC with a contractile phenotype more oxidative in tibialis muscle of p43−/− and Mstn−/− female mice, in the M16/pyruvate condition, was accompanied by a stimulation of the mitochondrial complex IV activity (Figure 6D,H). Taken together, these data confirm: (1) that the contractile phenotype of the skeletal muscle at adulthood is highly sensitive to a short exposure of pyruvate in the culture medium of the early embryo; and (2) that the phenotype is gender dependent.

### 2.7. Influence of Culture Medium at 1 Cell Embryo Stage on H3K27me and H3K4ac Marks

Our data show that a modification for a very short time of the early embryo environment has consequences on the adulthood phenotype in agreement with the DoHaD hypothesis. We can now ask if an early modulation of epigenetic marks could explain this later life alteration of skeletal muscle phenotypes observed. Recently, Nagaraj et al. [35] studied epigenetic reprogramming in 2-cell embryos cultured either in a medium (modified KSOM (potassium-supplemented simplex optimized medium)) lacking pyruvate, lactate, and glucose or in this mKSOM containing only pyruvate as energy source. They found that there is a strong decrease of H3K4 acetylation and H3K27 trimethylation levels in embryos cultured in carbohydrate-deprived medium compared to pyruvate alone medium. 

**Figure 7:**
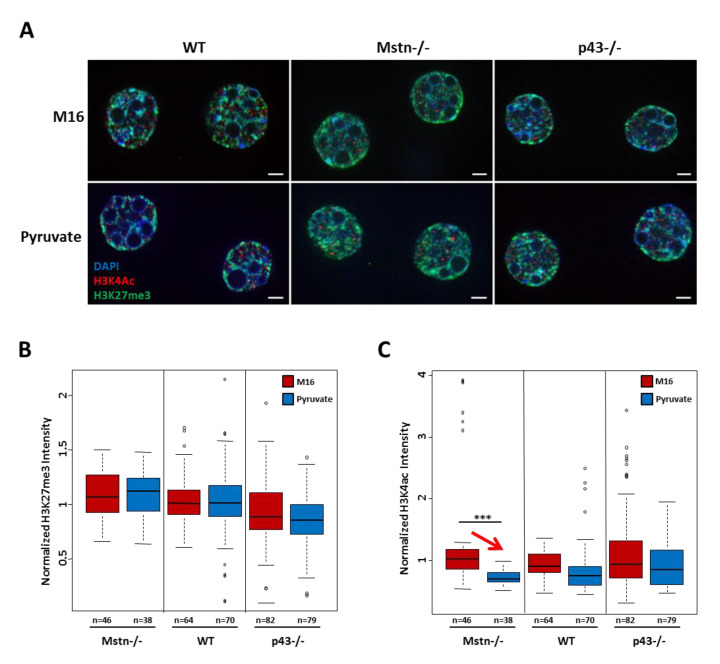
Culture medium containing pyruvate alone decreased H3K4 acetylation in Mstn−/− 2-cell embryo. (**A**) Representative staining of 2-cell embryos labelled with DAPI (4′,6-diamidino-2-phénylindole) and antibodies raised against H3K4Ac or H3K27me3. Scale bar: 5 µm. (**B**) Quantification of H3K27me3 fluorescent intensity normalized by DAPI fluorescent intensity. (**C**) Quantification of H3K4Ac fluorescent intensity normalized by DAPI fluorescent intensity. Red arrow indicates a significant decrease. For C57Bl6/6 nuclei, *n* = 64 in M16 medium and 70 in M16/pyruvate medium. For Mstn−/− nuclei, *n* = 46 in M16 medium and 48 in M16/pyruvate medium. For p43−/− nuclei, *n* = 82 in M16 medium and 79 in M16/pyruvate medium. Statistical Significance: *** *p* < 0.001, with non-parametric test using R project (Kruskal–Wallis). Results are expressed as ±sem.

On the basis of this work, we decided to quantify the H3K4 acetylation and H3K27 trimethylation levels with immunofluorescence experiments on 190 embryos cultured either in M16 or in M16/pyruvate media (Figure 7A). We found no significant difference for H3K27 trimethylation levels depending on the medium, whatever the genotype (Figure 7B). We observed a significant decrease of the mean intensity of fluorescence for H3K4 acetylation in Mstn−/−2-cell embryos (−47%, *p* < 0.001) exposed to M16/pyruvate medium. A slight but not significant decrease was also found in WT and p43−/− embryos (Figure 7C). Therefore, a brief exposure of 1-cell embryos to a medium containing pyruvate as the only source of carbohydrate has a modest influence on the acetylation level of H3K4 at 2-cell stage. Although subtle, these changes of an epigenetic mark are consistent with the hypothesis that early environmental changes can modify the epigenetic landscape and then adulthood phenotype.

## 3. Discussion

Early mammalian embryos utilize pyruvate, lactate, and/or amino acids as the major source of energy rather than glucose before the morula stage [36,37]. In this study, we investigated the influence on the adulthood phenotype of a transient change in the metabolism of 1 cell embryos. To this end, we compared a medium which contains pyruvate, lactate, and glucose as a carbohydrate energy source and a medium containing pyruvate as the only source of carbohydrate. Pyruvate goes into the embryo both passively and by means of a facilitated carrier [38]. Pyruvate alone in the medium is known to induce a transient NAD(P)H oxidation and stimulate mitochondrial activity [12]. Indeed, in Banrezes et al. (2011), the authors cultivated PN stage embryos in a medium with variation in carbohydrate contents (only lactate or only pyruvate or none of them). They used FADH2/FAD2+ ratio to measure mitochondrial activity and showed that, with pyruvate as the only source of carbohydrate, the FADH2/FAD2+ ratio is maintained, and the mitochondrial NAD(P)+ is increased [31]. These results are consistent with previous studies on mouse oocytes and embryos that demonstrated that pyruvate induces an increase of NAD(P)H oxidation and therefore stimulates mitochondrial activity [12,31]. In this work, more than 200 male and female mice were monitored and analyzed for up to 6 months. Of note the embryo production, the percentage of survival and the litter size were quite similar, whatever the genotype of the mice and the culture medium used (Figure S1). These data indicated that neither the genotype nor the pyruvate as the only energy substrate affect mouse embryo viability in our experiments. 

Interestingly, our study demonstrated that medium containing pyruvate as the only source of carbohydrate could induce an early orientation of the skeletal muscle contractile phenotype in C57Bl/6J mice, as well as in two mouse models of muscle hypertrophy. Furthermore, in most cases, we have shown that changes in the expression of MyHC were large enough to cause changes in the activity of the mitochondrial respiratory chain in the whole muscle. In addition, we found that these changes are in opposite directions depending on gender. For all male genotypes, a switch to a more glycolytic phenotype was recorded, whereas, in females, the pyruvate induced a switch to a more oxidative phenotype. Overall, these experiments underscore that the contractile and metabolic features of muscle, which are essential determinants of energy homeostasis, are highly sensitive to pyruvate in mouse. This observation is in line with recent studies highlighting that culture medium can influence postnatal growth, glucose metabolism, and vascular function in mouse models [12,18,23]. 

However, these changes in muscle mass seem to depend on the genotype and gender. In particular, the observation that pyruvate treatment in p43−/− male mice fully abolished the skeletal muscle hypertrophy compared to WT animals was very interesting and could explain the origin of the increase of muscle mass in p43−/− mice. Because the absence of p43 results in a decrease of the mitochondrial activity [7,39], these data suggest that the stimulation of mitochondrial activity induced by pyruvate treatment could restore a normal mitochondrial activity in p43−/− zygotes. In addition, the increase of muscle hypertrophy induced in Mstn−/− mice of both genders by pyruvate treatment was surprising because these mice are already very muscular. Our finding revealed that male and female offspring display different levels of sensitivity to pyruvate treatment and joined previous studies in stating that the gender difference is an intriguing feature of developmental programming [18,24,40]. This presumably reflects differences in their chromosomal complement and physiology [41]. 

Our experiments demonstrated that the presence of pyruvate alone in the medium, which mimics a stimulation of mitochondrial activity, plays a key role in the mouse embryo in the establishment of the skeletal muscle phenotype in adulthood. However, the link between the mitochondrial activity of the embryo, developmental programming, post-natal growth, and the long-term modulation of the muscular phenotype remains to be identified. Over the past decade, numerous microarray studies demonstrated that in vitro embryo manipulation (culture medium composition, oxygen tension, and method of fertilization) in mice influenced transcriptional profiles in blastocysts and in adult offspring tissues [26,42,43,44,45]. However, most of these studies were performed with embryos incubated for several days up to the blastocyst stage. Although, up to this stage (4–5 days), the embryos exhibit some cellular uniformity, this incubation window remains too long to precisely target the molecular events likely to be at the origin of the long-term influences. In this regard, our data demonstrate that transient incubation of embryos at the one cell stage is sufficient to induce long-term consequences and suggest that specific epigenetic modifications could occur and affect the accessibility of genomic regions, such as promoters, preventing or allowing the binding of transcription factors. Histones, which composed the nucleosome, are major actors of these epigenetic regulations and therefore play a central role in transcription regulation, DNA repair, DNA replication, and chromosomal stability [27]. Thus, histone 3 (H3) post-translational modification, such as acetylation and methylation are involved in regulation of genes expression [29,30]. Nagaraj et al. [35] found that several post-translational modifications of histone H3 were differentially regulated in 2-cell embryo cultured either in a medium lacking pyruvate, lactate, and glucose or in medium containing only pyruvate as energy source. Taking advantage of these results, we explored two of these marks namely H3K4ac and H3K27me3. However, in our conditions, despite the analysis of several hundred of nuclei, no change in H3K27 trimethylation were revealed, but a slight decrease of the H3K4 acetylation levels was observed in Mstn−/− 2-cell embryos cultured with pyruvate alone. This last finding is in agreement with previous work of Nagaraj et al. [35]. 

Genome wide reprogramming of histone modifications involving several enzymes (such as Histone demethylase and Histone acetyltransferase) occurs during the two first cell cycles of mouse development, leading to embryonic genome activation [46]. This major process needs availability of metabolites, such as Acetyl-CoA or alpha-ketoglutarate, which are co-factors or substrates of these enzymes. Accordingly, there is a tight interconnection between epigenetic and metabolism pathways [47,48]. 

Nagaraj et al. demonstrated that the pyruvate deshydrogenase complex translocates in the nucleus of the 2-cell stage embryos to produce Acetyl-CoA with pyruvate. In a medium without lactate or glucose but only pyruvate as energy source, we can hypothesize that pyruvate would also be used (i) in the mitochondria to provide TCA cycle with Acetyl-CoA and (ii) in the cytosol to produce lactate and then ATP in an anabolic manner [49]. Therefore, we can explain a decrease of H3K4ac levels by a decrease of pyruvate availability in the nucleus which in turn decreases the availability of Acetyl-CoA for the histone acetyltransferase. Finally, in a Mstn−/− genotype, a higher histone deacetylation may be explained with an increase of NAD+ availability for sirtuins that are NAD+ dependent deacetylase [50]. 

Regarding H3K27 trimethylation, the lack of difference observed in our conditions, in contrary to Nagaraj et al.’s [35] results, could be explain by the fact that this study compared embryos cultured in medium with pyruvate alone to a carbohydrate-deprived medium, a more drastic condition which impairs embryonic viability [51]. We can assume that, in our conditions that did not affect the viability and development of the embryo, the epigenetic changes are probably more subtle. Given the cumbersome nature of the experiments, we did not look at a lot of different epigenetic marks, neither at several embryonic stages (morula or blastocyst) after the switch in the embryo metabolism [52]. These findings open up new post-translational modification of histone to explore in our culture conditions. 

Indeed, our experimental design could be an opportunity to assess the molecular and thus epigenetic mechanisms involved in the detection and then the transduction of environmental inputs into a programmed metabolic response according to the DOHaD hypothesis [11].

## 4. Materials and Methods

### 4.1. Animals and Ethics Statement

Mice were housed and maintained on a 12-h light/dark cycle (lights on at 7:30 a.m.). Food (A03, SAFE) and water were provided ad-libitum. All animal experiments were performed according to European directives (86/609/CEE and 2010/63/CEE) and approved by Région Ethics committee in animal testing of Languedoc-Roussillon (French National agreement CEEA-036) with the identification code (CEEA-LR-1209113001 issued on 03 March 2013). Our institution guidelines for the care and use of laboratory animals were observed, including environmental enrichment in each cage (nesting cotton squares). Our animal facility is approved by the Departmental Veterinary Services (No. E34-172-10) and French Ministry of Research (No. 7053, 26 February 2020). p43−/− mice, lacking specifically the mitochondrial T3 receptor p43 were generated in our team as described previously (Blanchet et al., 2012). Mstn−/− mice, harboring a constitutive deletion of the third myostatin exon, have been described previously and were generously provided by V. Blanquet (INRAE, UMR-GMA, University of Limoges, France). All mice are in C57Bl/6J background. Throughout the study, mice were given free access to food and tap water. According to the European Directive 2010-63-EU, mice were observed daily for the general health status and mortality scoring. Any obvious signs of disease, injury, and behavioral disorder indicating pain were recorded. If signs persist for more than 48 h, the animal was euthanized by cervical dislocation.

After 6 months, the mice were sacrificed. The different tissues (BAT, WAT, and liver) and muscles were weighed. The weight of BAT, WAT, and liver is expressed in g, and the weight of muscle is expressed in mg. 

### 4.2. Embryo Production, Collection, and Treatment

WT, p43−/−, and Mstn−/− mice were mated without superovulation of females. Zygotes were recovered, and only fertilized 1-cell embryos displaying two PN were subjected to experimental treatments. Two culture media were used: a standard M16 [53] that contains pyruvate, lactate, and glucose as energy source and a M16/pyruvate without lactate and glucose but only pyruvate (0.33 mM) as the only source of carbohydrate in the culture medium. NaCl concentration was increased in the M16/pyruvate medium to maintain osmolarity.

Fertilized 1-cell embryos were incubated for about 1 min in a hyaluronidase solution in M2 medium (Type IV-S from bovine testes, (Sigma-Aldrich, Saint-Quentin Fallavier, France) H4272, 1 mg/mL) to remove cumulus cells. Zygotes were rinsed thoroughly in M2 medium (Sigma-Aldrich) and placed in 500 µL drops of experimental media in a petri dish with central well certified for in vitro fertilization (Nunc). They were cultured for about 19 h in an atmosphere of 5% CO_2_ in air at 37 °C. The 2-cell embryos were then transferred to pseudo-pregnant recipients or fixed with 2% PFA for 20 min (RT) to do the immunostaining experiments.

### 4.3. Embryo Transfer and Post-Natal Growth

Female F1 mice (C57BL/6J × CBA/J), 8–24 weeks old, were used as 0.5-day p.c pseudo-pregnant recipients. Experimental and control eggs at the two-cell stage were transferred in groups of 8–10 into the ampulla of left oviduct of each pseudo-pregnant recipient female. Starting the first week after delivery, newborns were weighed every week for 6 months. The pups were weaned at the 4th week, and males and females in each litter were separated and placed in groups of 4 in independent cages. Mice were housed and maintained on a 12 h light/dark cycle. Food (AO3, SAFE diets, Augy, France) and water were provided ad-libitum. 

### 4.4. Gene Expression Studies

Total RNAs were extracted from tibialis muscle using Trizol^®^ and cDNAs were generated using the PrimeScript™ 1st strand cDNA Synthesis Kit (Takara Bio, Saint-Germain-en-Laye, France). Real-time PCR was performed using the SYBR^®^ Premix Ex Taq™ II (Takara Bio) and an Applied Biosystems Step One Plus (Thermo Fisher Scientific, Waltham, MA, USA). Gene expression was normalized to the expression of the housekeeping gene *Rps9* and is expressed as means ± sem. Student’s *t* test were used to determine all *p* values. Primer sequences have been described previously [8]. 

### 4.5. Mitochondrial Complex IV Activity

Tibialis muscle homogenates were prepared at 4 °C in 1 ml phosphate buffer (50 mm; pH 7) using a Polytron homogenizer. Mitochondrial complex IV activity (Cytochrome c oxidase) was measured spectrophotometrically by following the oxidation of reduced cytochrome c at 550 nm for 30 s [54]. Proteins concentration was measured using the Bio-Rad (Hercules, CA, USA) protein assays kit.

### 4.6. Immunostaining of Post-Translational Modifications of Histones and Fluorescence Intensity Measurements

After a short rinse in PBS, 2-cell embryos were permeabilized at room temperature in 0.5% Triton X-100 (Sigma-Aldrich) for 30 min. They were then transferred in a blocking solution (2% BSA, Sigma-Aldrich) for at least 30 min at room temperature (RT) before incubation, at 4 °C overnight, in primary antibodies diluted in 2% BSA. After three rinses in PBS for 10 min at RT, embryos were incubated with secondary antibodies coupled to fluorophores diluted in 2% BSA for 1 h (RT). The antibody solution was then rinsed with PBS at least three times (10 min, RT) and mounted between slides and cover slips in Vectashield (Vector Laboratories, Burlingame, CA, USA) containing DAPI (1:100 of a solution at 1 mg/ml, Sigma-Aldrich). The following antibodies were used in this study: rabbit polyclonal antibody against H3K4 acetylation (Abcam, Cambridge, UK) (ab176799) at 1:500 dilution and a mouse monoclonal antibody against H3K27 di/trimethylation (Active motif, Carlsbad, CA, USA) (39536) at 1:250 dilution. These primary antibodies were revealed with secondary antibodies at 1:200 dilution purchased from Jackson Immunoresearch (Cambridge, UK) against Rabbit (JIR 711-165-152) coupled with Cy3 or Mouse (JIR 715-095-151) couple with FITC. 

The embryos were examined with a Zeiss Axiovert Apotome microscope (microscopy and imaging platform MIMA2, Jouy-en-Josas, INRAE) with a x63 oil-immersed lens objective (N.A.1.4). Stack images were acquired with a z-step of 0.5 µm and a frame size of 1348 × 1040 pixel depth. LED wavelengths of 365 nm, 470 nm, and 555 nm allow us to observe, respectively, DAPI (DNA), FITC (H3K27me3), and Cy3 (H3K4ac) fluorescent staining in three distinct channels. 

Intensity of fluorescence in the nucleus was quantified using the ImageJ software following the next procedure. After a z-projection by summing slice intensity of all stacks containing the 2-cell embryo, regions of interest (ROI) of each nucleus and of a large area dedicated to background measurement were drawn. Measurements of the area and the mean intensity were done for each ROI. Fluorescent background was then subtracted from the measure of the total intensity in a nucleus for a dedicated channel (DNA, H3K27me3, and H3K4ac) using this formula = ((mean of fluorescence intensity measured for a Channel) − (mean of fluorescence intensity measured for the BG_channel_)) × Area of the ROI_nucleus_ measured). This "total amount of fluorescence intensity" was then divided by the exposure time (ms). To be able to compare this value calculated for H3K27me3, H3K4ac, and DNA between several embryos from different experiments and genotypes, we also normalized it by dividing each channel, respectively, by the "mean amount of fluorescent intensity" of the WT embryos in M16 condition (we call it thereafter the "normalized fluorescence"). Then, to get rid of a putative variation of the amount of DNA during the cell cycle, we also calculated a ratio that correspond to the normalized fluorescence of a histone marks divided by the normalized fluorescence of the DNA for each nucleus in each condition / genotype. The numbers of nucleus analyzed in each group (WT, Mnst−/−, and p43−/−) are reported in the legend of the Figure 7.

### 4.7. Statistical Analyses

All results are presented as means ± sem, or as percentages. Statistical significances of the differences between groups were evaluated with Student’s t-test or with non-parametric test using R project (R Core Team (2014); R: A language and environment for statistical computing. R Foundation for Statistical Computing, Vienna, Austria. URL http://www.R-project.org/) (“nparcomp” package) [50].

## 5. Conclusions

**Figure 8:**
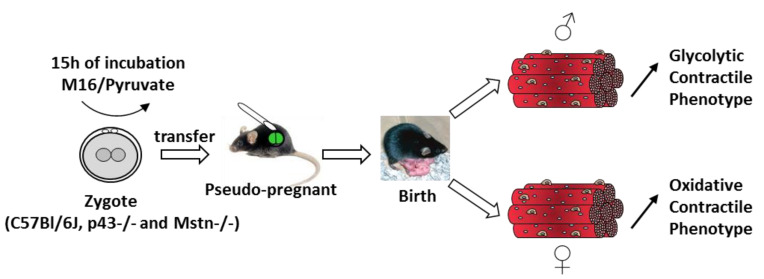
Schematic representation of the influence of pyruvate treatment of embryo at one cell stage on skeletal muscle phenotype.

In conclusion, to our knowledge, we are the first to report that a transient perturbation of the metabolism of a 1 cell embryo induces prolonged effects in the adult phenotype in C57Bl/6J mice, as well as in two mouse models of muscle hypertrophy. In particular, our findings indicate that pyruvate alone affects contractile and metabolic skeletal muscle phenotype in opposite ways depending on gender. However, some changes in muscle mass seem to depend on the genotype. Overall, these findings demonstrate that early stage embryo metabolism can induce an early orientation of skeletal muscle phenotype observed in adulthood (Figure 8). It will be very interesting to determine whether the process outlined here for the skeletal muscle phenotype have implications for breeding animals better adapted to climate change. It will be also interesting to determine whether these observations have clinical relevance for infertility research, including in vitro fertilization and other assisted reproductive technologies procedures. 

## Supplementary Materials

Supplementary Materials can be found at https://www.mdpi.com/1422-0067/21/19/7203/s1. Figure S1. (A) Developmental potential of experimental eggs. (B) Mean litter size in vivo, in M16 medium or in M16/Pyruvate medium. Number of litters analyzed: C57BL6 in vivo (*n* = 50), C57BL6 M16 (*n* = 13), C57BL6 pyruvate (*n* = 6), p43−/− in vivo (*n* = 50), p43−/− M16 (*n* = 7), p43−/− pyruvate (*n* = 5), Mstn−/− in vivo (*n* = 50), Mstn−/− M16 (*n* = 5), Mstn−/− pyruvate (*n* = 6). Statistical Significance: * *p* < 0.05; ** *p* < 0.01, Student’s *t*-test. Results are expressed as ±sem. Figure S2. (A and B) Post-natal growth profiles of male (A) and female (B) C57Bl/6J, p43−/− and Mstn−/− mice derived from zygotes incubated in M16 medium. Statistical Significance: *** *p* < 0.001, Student’s *t*-test. Results are expressed as ±sem. Figure S3. (A–D) Comparison of tissue weights of male and female from C57Bl6, p43−/− and Mstn−/− mice derived from zygotes incubated in M16 medium at 6 months of age. Statistical significance: * *p* < 0.05, ** *p* < 0.01, *** *p* < 0.001, Student’s *t*-test. Results are expressed as ±sem. Figure S4. (A and C) Relative mRNA expression levels of the four Myosin Heavy Chain (MyHC) isoforms in tibialis muscle of male (A) and female (C) from C57Bl6, p43−/− and Mstn−/− mice derived from zygotes incubated in M16 media. (B and D) Mitochondrial complex IV activity (cytochrome c oxidase) in tibialis muscle of male (B) and female (D) from C57Bl6, p43−/− and Mstn−/− mice derived from zygotes incubated in M16 medium at 6 months of age (*n* = 8 each group). Statistical significance: * *p* < 0.05, ** *p* < 0.01, *** *p* < 0.001, Student’s *t*-test. Results are expressed as ±sem.
