# Supplementary material for: Transient Changes of Metabolism at the Pronuclear Stage in Mice Influences Skeletal Muscle Phenotype in Adulthood
Source: Int J Mol Sci. 2020 Sep 29;21(19):7203. doi: 10.3390/ijms21197203 (PMC7582979; doi:10.3390/ijms21197203)
Supplement: Supplementary file 1 [file ijms-21-07203-s001.pdf]

A

Treatment	# Egg	# 2-C (%)	# Recipient	# 2-C transferred	# Viable Pup	Survival (%)	Litter size±SEM
C57Bl6 / M16	207	92	14	114	63	55	5,2±0.5
C57Bl6 / Pyr	91	88	6	46	33	72	5.5±0.6
p43-/- / M16	125	90	8	70	39	56	5,6±0.5
P43-/- / Pyr	64	89	5	45	30	67	6±0.5
Mstn-/- / M16	69	92	6	52	28	54	5.6±1.3
Mstn-/- / Pyr	93	85	7	56	31	55	5,2±0.8
Total	649	90 %	46	383	231	61	5.4±0.3

B

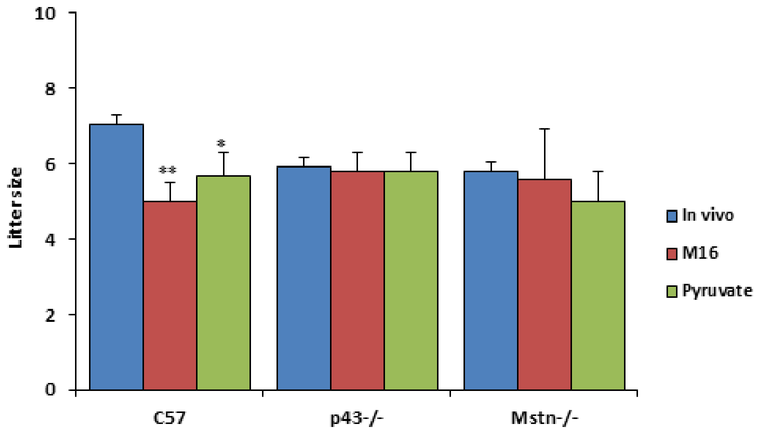

Figure S1 (**A**) Developmental potential of experimental eggs. (**B**) Mean litter size in vivo, in M16 medium or in M16/Pyruvate medium. Number of litters analyzed: C57BL6 in vivo (*n* = 50), C57BL6 M16 (*n* = 13), C57BL6 pyruvate (*n* = 6), p43-/- in vivo (*n* = 50), p43-/- M16 (*n* = 7), p43-/- pyruvate (*n* = 5), Mstn-/- in vivo (*n* = 50), Mstn-/- M16 (*n* = 5), Mstn-/- pyruvate (*n* = 6). Statistical Significance: *$p < 0.05$; **$p < 0.01$, Student's t-test. Results are expressed as ± sem.

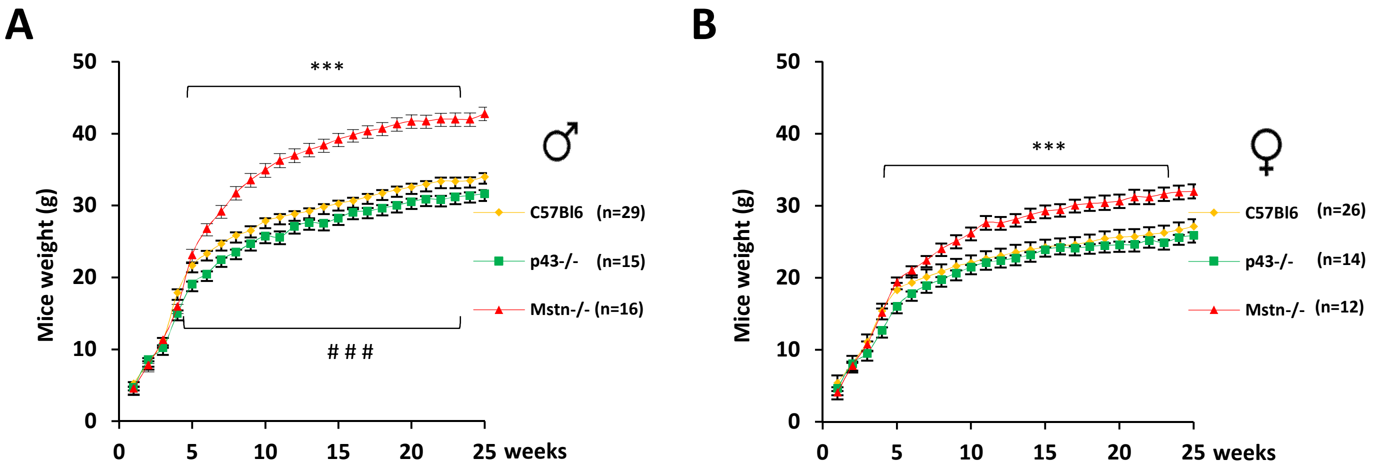

Figure S2 (**A** and **B**) Post-natal growth profiles of male (**A**) and female (**B**) C57Bl/6J, p43-/- and Mstn-/- mice derived from zygotes incubated in M16 medium. Statistical Significance: ***$p <$ 0.001, Student's *t*-test. Results are expressed as ± sem.

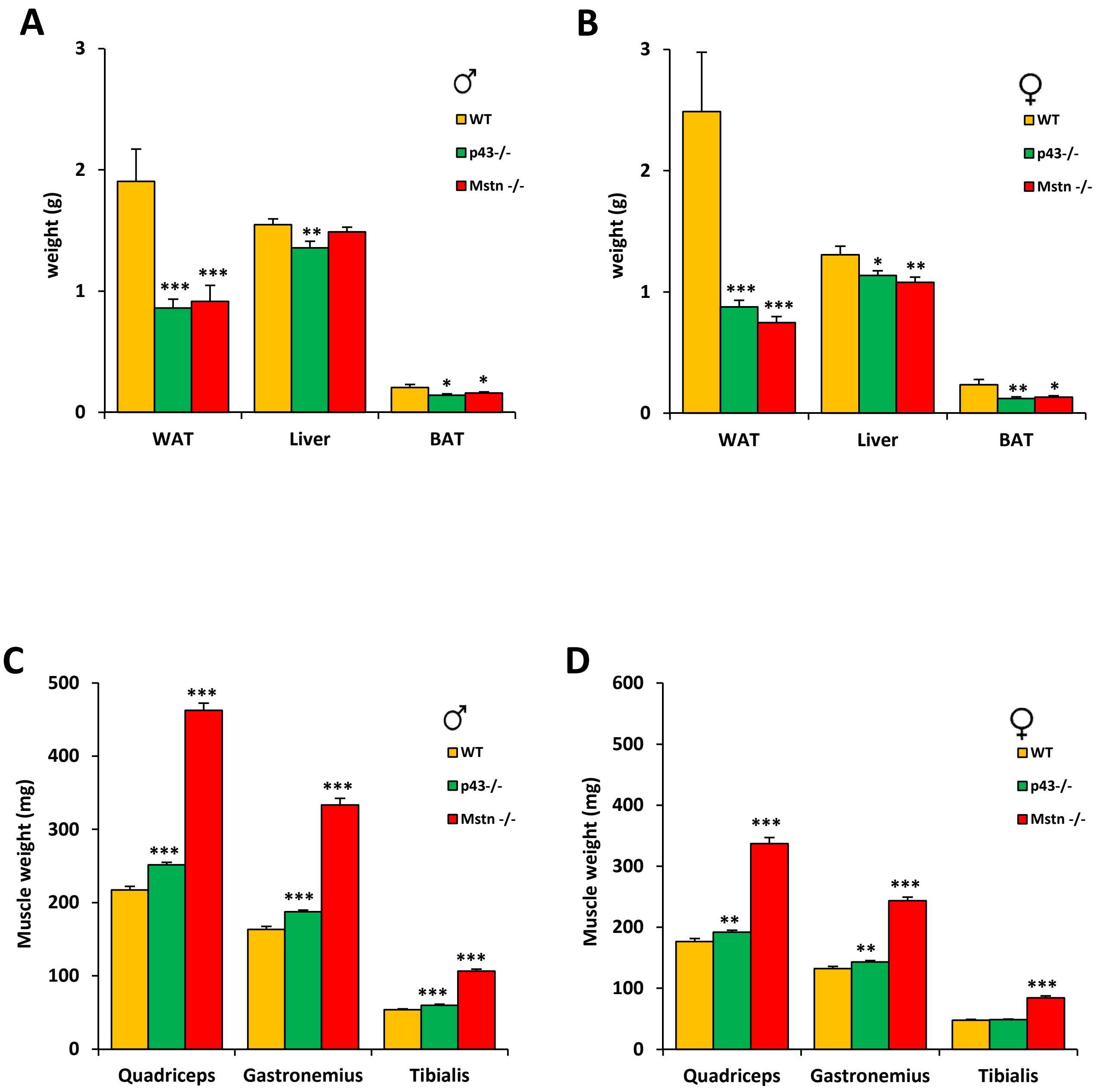

Figure S3 (**A–D**) Comparison of tissue weights of male and female from C57Bl6, p43-/- and Mstn-/- mice derived from zygotes incubated in M16 medium at 6 months of age. Statistical significance: *$p < 0.05$, **$p < 0.01$, ***$p < 0.001$, Student's *t*-test. Results are expressed as ± sem.

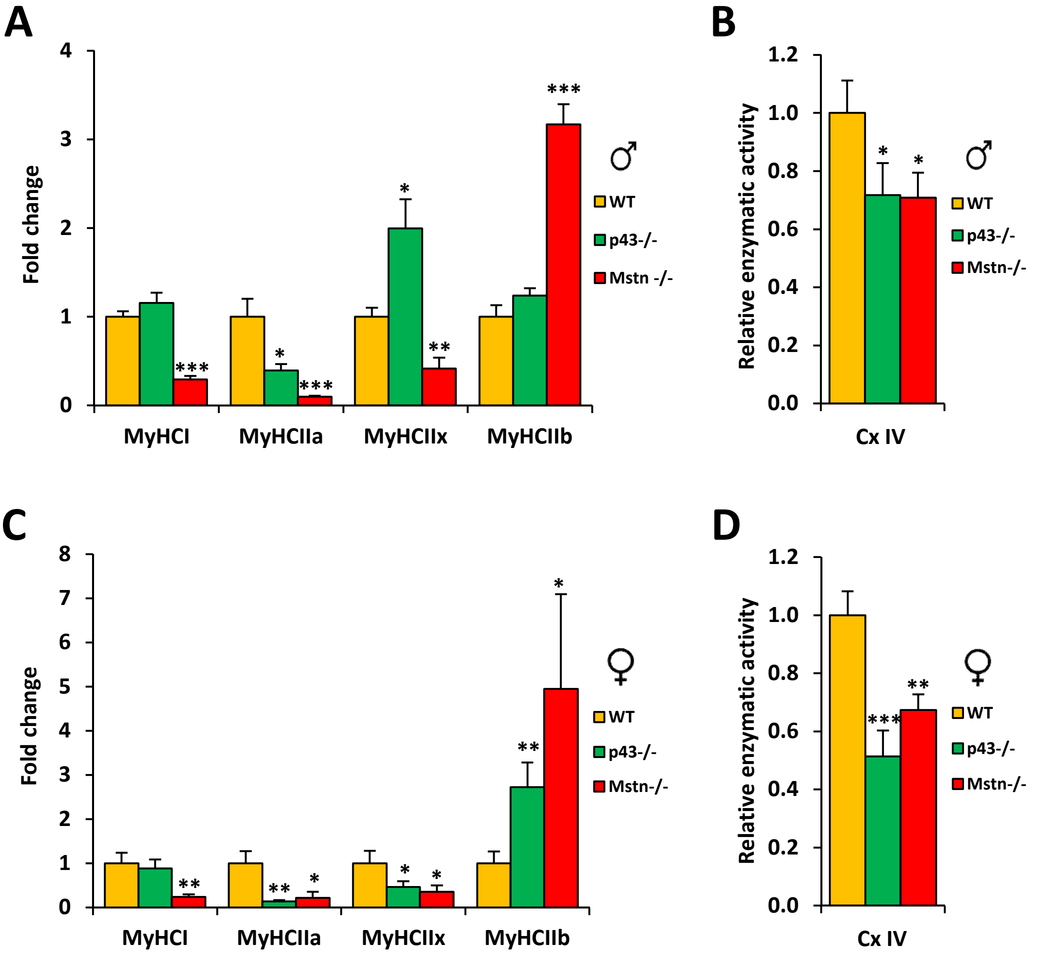

Figure S4 (**A** and **C**) Relative mRNA expression levels of the four Myosin Heavy Chain (MyHC) isoforms in tibialis muscle of male (**A**) and female (**C**) from C57Bl6, p43-/- and Mstn-/- mice derived from zygotes incubated in M16 media. (B and D) Mitochondrial complex IV activity (cytochrome c oxidase) in tibialis muscle of male (**B**) and female (**D**) from C57Bl6, p43-/- and Mstn-/- mice derived from zygotes incubated in M16 medium at 6 months of age (n = 8 each group). Statistical significance: *$p < 0.05$, **$p < 0.01$, ***$p < 0.001$, Student's t-test. Results are expressed as ± sem.
